# A review of multiparametric ultrasound imaging in the clinical setting: scrotal contents

**DOI:** 10.1007/s00261-024-04587-z

**Published:** 2024-09-19

**Authors:** Paul S. Sidhu, Gibran T. Yusuf, Maria E. Sellars, Annamaria Deganello, Cheng Fang, Dean Y. H. Huang

**Affiliations:** 1 King’s College London, London, UK; 2 King’s College Hospital, NHS Foundation Trust, London, UK

**Keywords:** Ultrasound, Contrast ultrasound, Testis, Scrotum, Elastography

## Abstract

The innovative techniques in ultrasound have added a new dimension to investigating superficially located areas such as the contents of the scrotal sac. High frequency transducers, improved technology with the addition of elastography, contrast enhanced ultrasound and microvascular imaging has resulted in a further improvement in diagnostic capabilities. The ability to clearly demonstrate the presence or absence of vascularity within the area under investigation adds an additional dimension to operator confidence in establishing the presence of infarction, global or segmental, or the walls and cavity of an abscess in the testis or epididymis. Increased vascularity of a tumor aids the differential diagnosis based on the flow dynamics of the microbubble contrast, benign lesions likely to retain contrast. Elastography has the ability to ascertain the stiffness of tissue, and when used in conjunction with other ultrasound methods adds to the understanding of the likelihood of a malignant abnormality being present. All the different techniques come under the umbrella term ‘multiparametric ultrasound’, with the application in the scrotal sac detailed in this article.

## Introduction

The ability of ultrasound to assess the superficial structure is unique, with high frequency transducers providing high resolution imaging, with recent advances in transducer technology coupled with advances in ultrasound techniques, termed multiparametric ultrasound, improving the imaging capabilities [1], transforming the ability to assess scrotal contents. High frequency, and with it better resolution, combined with broadband capabilities renders ultrasound assessment of scrotal contents the first line and often only imaging technique needed for diagnosis. Earlier addition of color and spectral Doppler techniques to the conventional B-mode ultrasound, were useful in assessing vascularity of focal lesions, inflammation and the extent of vascularity in the traumatized testis and added important information to the examination, particularly in assessing epididymal inflammation and the possibility of spermatic cord torsion [2–4]. Newer ultrasound techniques, including microvascular imaging (MVI) [5] elastography [6, 7] and contrast enhanced ultrasound (CEUS) [8] has further improved capabilities for the assessment of the scrotum [9]. Recent guidelines for the use of these newer ultrasound techniques, allows for the incorporation into clinical practice for better patient care [10–12].

The article will describe the application of ultrasound to the diagnosis of scrotal disease, by assessing presenting symptoms and clinical scenarios, incorporating new ultrasound techniques to allow for confident diagnosis and patient management without recourse to other imaging techniques. The use of all these methods in an ultrasound examination is ascribed to an umbrella term of multiparametric ultrasound (MPUS) [1].

## Newer techniques in ultrasound

### Contrast enhanced ultrasound

The most widely used microbubble contrast agent for the assessment of scrotal disease is Lumason™/SonoVue™ (Bracco SpA, Milan), used off license when applied to the scrotal contents but with justification and supported by regulatory authorities, when beneficial to the patient [13]. The examination requires a full dose of the microbubble agent (normally 4.8mls of Lumason™/SonoVue™), as the physics of the harmonic response of the microbubble is governed by the acoustic properties in the insonating ultrasound field, with the smaller microbubbles required to interact with the higher frequency beam found at a smaller concentration in the injected dose [14, 15]. The technique is a natural extension of a liver CEUS examination, with the advantage of absence of movement of the scrotal contents. The important aspect of a CEUS examination in the scrotum is the arterial phase, with meaningful information obtained within 2 min of injection. With new software available the possibility to use multiple intensity projection methods of summation of the signal is useful, as often the intensity of the enhancement in the non-inflamed testis is limited. The appraisal of the vascularity of a focal intra-testicular lesion often requires the addition of time intensity curves for assessment of the perfusion and washout of these lesions [16, 17]. The ability to distinguish vascularized from non-vascularized components of the scrotal contents is key to the success of a CEUS examination, often improving the diagnostic yield of the ultrasound examination as establishing avascular tissue is paramount for the interpretation [11, 12]. Areas of infarction or abscess content will be readily identified, as absence of flow is clearly depicted. The excellent capabilities of CEUS to depict vascular flow to the capillary level combined with the novelty of being a truly intravascular contrast agent without leaking into the extra-vascular space, a hallmark of other imaging contrast agents, allows for confidence to interpret absent flow.

### Elastography

The ability to assess the stiffness of tissue is the basis of tissue elastography, and represents the “surgeon’s” hand when assessing scrotal abnormalities. The hallmark of malignant tissue is increased stiffness, potentially allowing for the differentiation of benign and malignant lesions within the testis parenchyma and out with the clinical examination. There are two methods of elastography in clinical use. Strain elastography, where pressure applied by the transducer is converted to a color map of stiffness, with a strain ratio applied between normal and abnormal tissue. The other method, shear wave elastography, which does not require external transducer pressure, has predominantly been used in the assessment of liver stiffness as a measure of liver fibrosis [18]. With shear wave elastography the ultrasound transducer generates a shear wave which can quantify stiffness in m/sec or kPa. This can be quantified using point shear wave elastography or 2-D elastography where a volume measurement is obtained, color coded, and region of interest drawn at the site of measurement [10] Elastography measurements, well established in assessing liver disease, are commonly used clinically in the assessment of breast and thyroid abnormalities [10, 19]. The use of strain elastography color maps have been investigated more thoroughly in the assessment of intra-testicular lesions, whereas the measurement of shear wave velocities have been used predominantly in the testis parenchyma in infertility [20, 21]. There is no specific advantage of either technique, with color mapping giving a visual overview, which aids interpretation. There is considerable overlap in the reported values for the measurement of stiffness in different testicular conditions, with a reported range for the normal testis of between 2.0 and 2.9 kPa (median 2.4 kPa) and oligoasthenoteratospermia between 1.8 and 2.5 kPa (median 2.1 kPa) [22].

s.

### Microvascular imaging

The development of Doppler US techniques has developed considerably, with the newest technique of increased sensitivity with reduction of artifacts, termed microvascular imaging, showing great promise in many areas [5]. Applications in the scrotal sac are not well documented, but with potential in many areas where vascularity is paramount to the diagnosis. This may be a useful tool when evaluating the presence of vascularity in a focal intra-testicular lesion, prior to the application of microbubble contrast. Movement artifact is an issue with microvascular techniques, with interrogation of the scrotal sac less problematic with absence of respiratory movement and a superficial location.

## Clinical applications

A description of the various abnormalities in the scrotal sac suitable for a multiparametric ultrasound examination will be detailed according to clinical scenarios. The European Federation of Societies for Ultrasound in Medicine and Biology (EFSUMB) guidelines on the use of CEUS for the testis, limits the application to some well-defined investigations; (i) detailing the presence or absence of vascularity in focal testicular tumors, (ii) ascertaining viable tissue in trauma, (iii) detailing the extent of a segmental infarction and (iv) ascertaining the configuration and site of an abscess [11]. Tissue elastography has a limited role as a stand-alone technique in the testis, and EFSUMB recommends that this technique should be used in conjunction with other ultrasound techniques when evaluating testicular abnormalities [10].

### Scrotal pain

Scrotal pain may be chronic, low level and often related to the presence of a varicocele, or acute and presenting as a surgical emergency, with spermatic cord torsion a consideration in the appropriate clinical setting. The patients age is crucial to the underlying cause, as is a detailed clinical history. Testicular tumors rarely present with pain, although this will be the anxiety of the person presenting with symptoms. Ultrasound is the imaging technique of choice and the addition of the many newly available techniques improve the diagnosis and management.

### Spermatic cord torsion

**Fig. 1:**
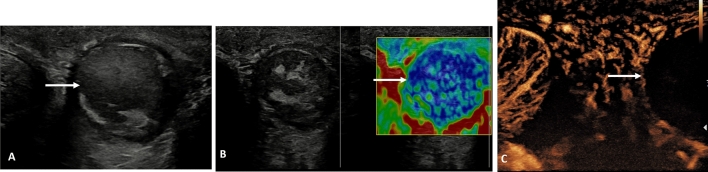
Presentation following several days of testicular pain in an 18 year old; spermatic cord torsion. **A** B-mode transverse view of the testis, demonstrating an enlarged heterogenous left testis (arrow). **B** Tissue elastography demonstrates a heterogenous color map, predominantly blue, which indicates increased stiffness (arrow). **C** A contrast enhanced ultrasound examination, using maximum intensity projection, confirms absence of flow in the left testis (arrow), confirming a “missed” spermatic cord torsion

This most often presents at the onset of puberty as the testicular volume increases, and is thought to be associated with the bell clapper deformity, although the prevalence of this deformity is far higher than the incidence of spermatic cord torsion [23]. The hallmark of the clinical symptoms is the sudden onset of pain, which is relentless, and constitutes a surgical emergency [24]. The time dependent testis salvage rate requires emergent surgery, and unless ultrasound is readily available, imaging should not delay surgery. Ultrasound is useful in determining the absence or presence of testicular color Doppler flow, or the presence of epididymitis or orchitis [25–27]. Absence of flow on color Doppler is considered the “standard” for the presence of a spermatic cord torsion, but this has a number of pitfalls; observer experience, ability to perform the examination accurately in the presence of severe pain, technical limitations of the equipment and importantly, intermittent spermatic cord torsion may present with normal color Doppler flow [3]. The addition of CEUS alters the confidence in establishing the absence of flow; areas of infarction will be clearly depicted [28]. The CEUS examination is particularly useful for a “missed” torsion when the patient presents days after the acute episode, with diminished pain, but an enlarged heterogenous testis, with poor color Doppler signal; absence of flow is unequivocal on CEUS [29]. The addition of tissue elastography in the pathway for assessing spermatic cord torsion has limitations and varies according to the duration of ischemic changes, but may have a role in the acute stages when there is intermittent blood flow compromise [30] (Fig. 1).

### Segmental infarction

**Fig. 2:**
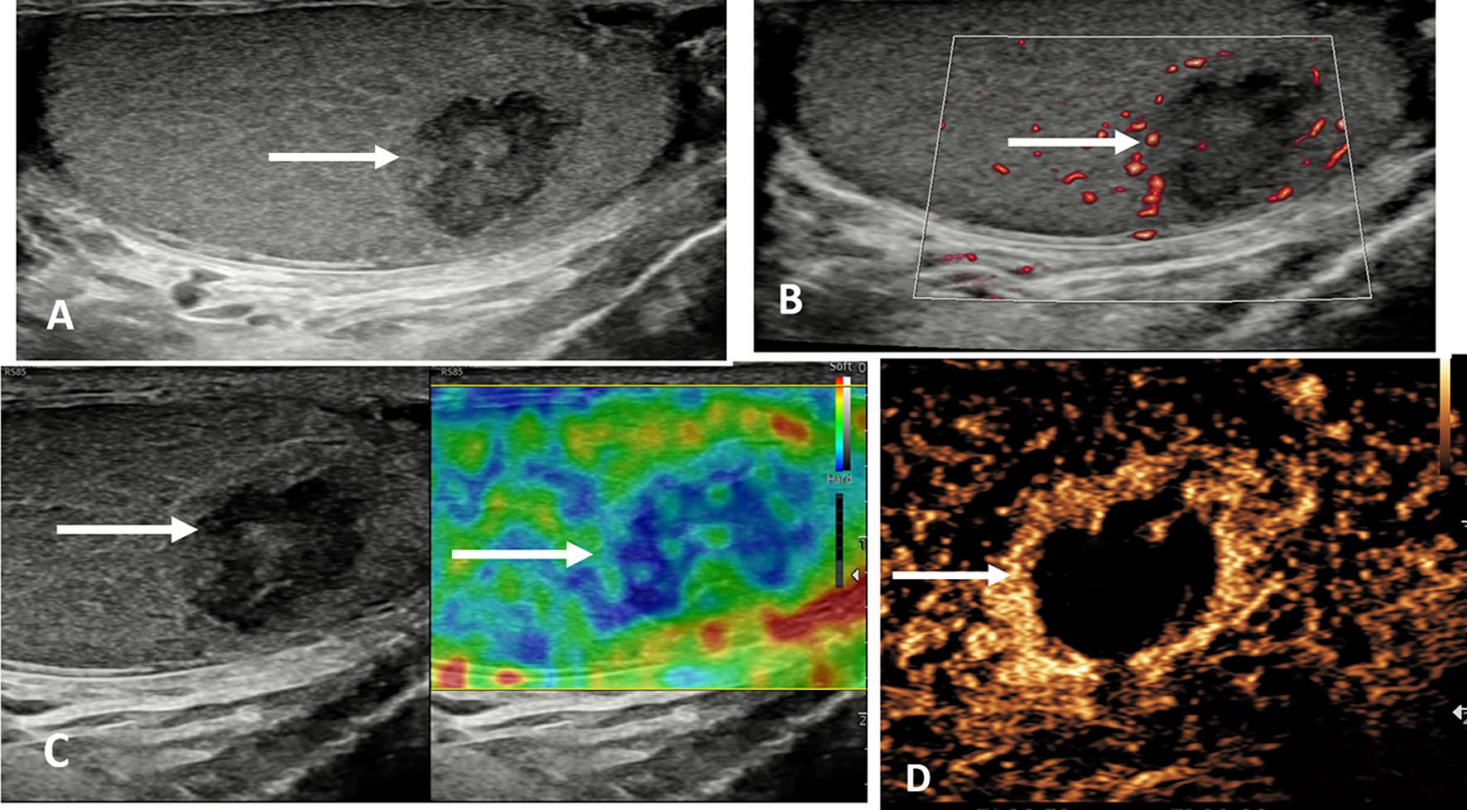
Presentation with acute testicular discomfort in a 62 year old; segmental testicular infarction. **A** B-mode longitudinal view of the left testis demonstrating a irregular focal lesion in the lower aspect of the testis (arrow). **B** Microvascular imaging shows color surrounding the lesion with possible internal faint color Doppler flow (arrow). **C** Tissue elastography demonstrates a mixed stiffness lesion, predominantly of increased stiffness (arrow); this will evolve over time. **D** The contrast enhanced ultrasound examination confirms an avascular lesion with surrounding hyperemia (arrow)

**Fig. 3:**
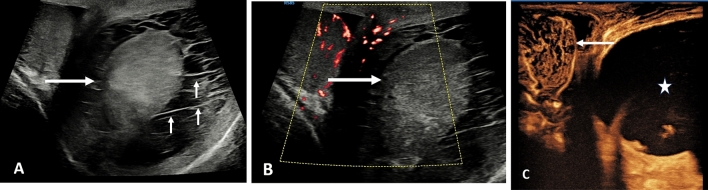
Acute and severe epididymitis and venous infarction of the left testis in a 58 year old. **A** The B-mode transvers view demonstrates the left testis (arrow) with a septations in a pyocoele (small arrows) in a patient with severe pain and underlying epididymitis. **B** The microvascular imaging image does not demonstrate any color flow in the left testis (arrow). **C** On the contrast enhanced ultrasound examination, with a maximum intensity projection, the right testis is adequately perfused (arrow) whilst the left testis (star) has no perfusion following global infarction associated with severe epididymitis

An area of segmental infarction in the testis has no specific pre-disposing factors, may be related to infection, sickle cell disease, vasculitis or idiopathic, affecting an older age group than that of spermatic cord torsion [4, 31]. Color Doppler flow is normally absent, and visualized more accurately on MVI, but clearly depicted on the CEUS examination [32, 33] (Fig. 2). In the presence of severe epididymitis, venous drainage from the testis may be compromised, resulting in areas of segmental infarction, and in some severe cases, complete testicular infarction [34, 35] (Fig. 3). Conservative clinical management is advocated for areas of segmental infarction, early use of CEUS will allow confident diagnosis with follow-up advocated when areas of avascular tissue on CEUS reason against the presence of tumor, and elastography changes will mimic the changes in tissue stiffness over a time period [33].

### Infection

**Fig. 4:**
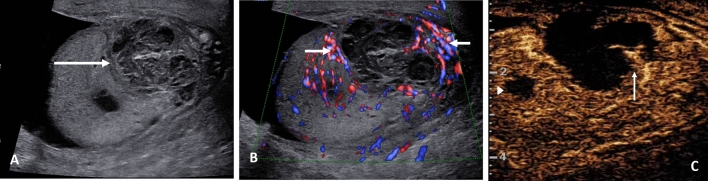
Focal abscess in the right testis in a 42 year old, following severe epididymitis. **A** Oblique view on B-mode ultrasound demonstrates a heterogenous focal lesion in the inferior aspect of the right testis (arrow). **B** Color Doppler ultrasound demonstrates an avascular area with some increased flow around the peripheral aspect of the lesion (short arrows). **C** Contrast enhanced ultrasound demonstrates the abscess cavity as an avascular area, with enhancement of a septation (long arrow). There is a second pocket of abscess formation demonstrated (arrowhead)

The presence of uncomplicated epididymitis is a clinical diagnosis and readily responds to medical treatment without resorting to imaging. Intractable symptoms whist on treatment warrants imaging [36]. The ability to differentiate orchitis, epididymitis and epididymis-orchitis is readily ascertained on ultrasound imaging and may alter management strategy [37]. Complications that arise that benefit from MPUS include abscess formation, both within the epididymis or in an intra-testicular location (Fig. 4), segmental infarction and global venous infarction [38, 39]. Color Doppler ultrasound and MVI will depict the hyperemic borders of an abscess, with an echo-poor center adequately, but the addition of CEUS will depict clear separation of viable and non-viable tissue, and allow for serial follow-up to resolution or expediate surgical intervention. Septations within an abscess cavity or within a chronic pyocoele may be seen with a CEUS examination. The presence of an echogenic pyocoele may obscure the underlying testis, and testicular viability can be assessed using CEUS. Serial elastography of an abscess will depict changes that vary with the resolution, and liquefication will be recognized on elastography with the classic ‘blue-green-red’ sign [40, 41]. Orchitis demonstrates a variable appearance on ultrasound, often with areas of low reflectivity, as infarcted areas interspersed with viable infective parenchyma [42], with CEUS and MVI adding further vascular information, and elastography demonstrating increased testis stiffness. An ‘end-stage’ complication of severe epididymitis is the development of venous outflow obstruction, a consequence of oedema and occlusion of the veins draining the testis, resulting in global venous testicular infarction [35].

### Torsion of an appendix testis

There are four testicular appendages present, two of which are visible on an ultrasound examination; the testis appendix and the epididymal appendix, with variation in size and appearance, and when cystic is known as the Hydatid of Morgagni [43]. Torsion of an appendix usually occurs in the pre-adolescent boy, and is an important differential for acute testicular pain, as management is conservative [44, 45]. Although color Doppler is used to ascertain loss of blood supply, CEUS has been reported in a case series to be of benefit to ascertain the perfusion to improve imaging diagnosis [46].

## Scrotal lumps 

### Intratesticular tumors

**Fig. 5:**
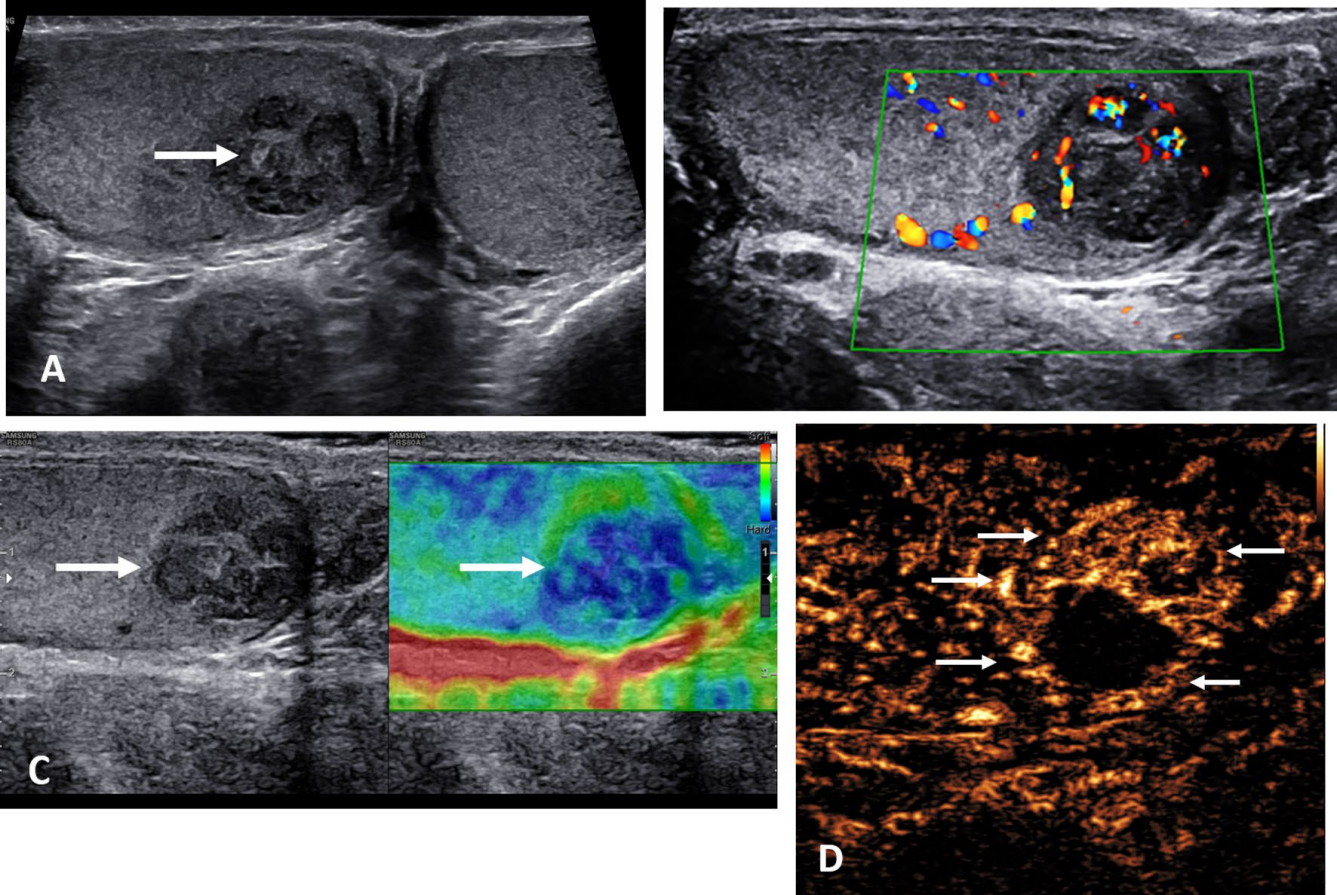
Stage 1 seminoma in a 51-year old. **A** Transverse view B-mode ultrasound of the testes demonstrating afocal heterogenous lesion in the right testis (arrow). **B** Longitudinal color Doppler view of the lesion showing intra-lesional color Doppler flow (arrows). **C** Tissue elastography demonstrates increase stiffness of the lesion, which is predominantly blue (arrows). **D** A contrast enhanced ultrasound examination demonstrating enhancement of the focal lesion (arrows) with a pocket of avascularity corresponding to an area of tumor necrosis

**Fig. 6:**
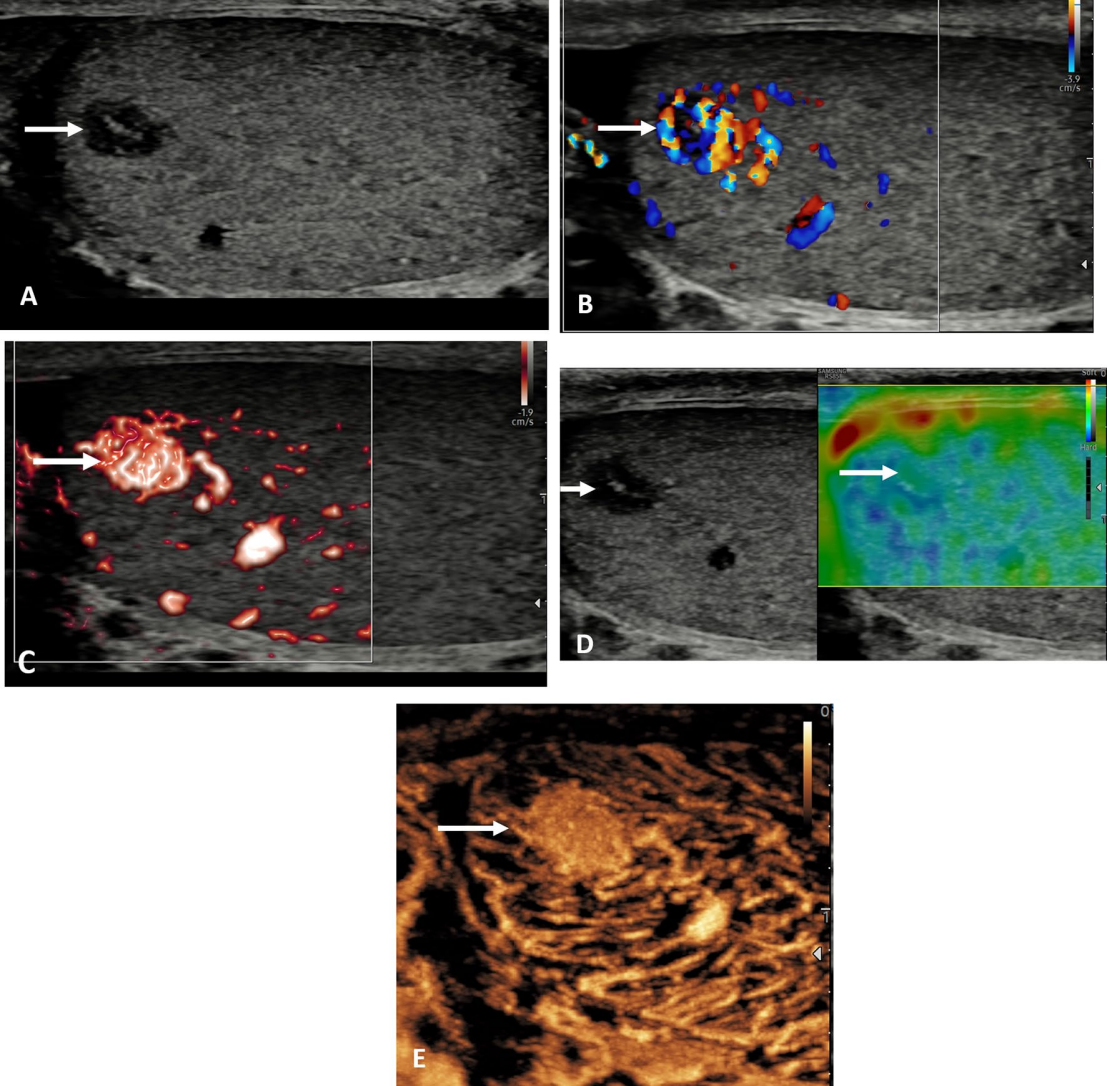
Incidental lesion in a 54 year old imaged for non-specific testicular pain. Testis sparing surgery with excision of the lesion showed Leydig cell hyperplasia. **A** A longitudinal image of the right testis demonstrating a 4 mm low reflective lesion (arrow). **B** The color Doppler image indicates increased vascularity of the focal lesion (arrow). **C** On the microvascular imaging there is intense vascularization present (arrow). **D** The tissue elastography image shows that the lesion is not stiffer than the surrounding normal tissue (arrows). **E** A contrast enhanced ultrasound examination, with maximum intensity projection, demonstrates a highly vascular lesion (arrow) that retains microbubble contrast at 90 s post contrast injection

**Fig. 7:**
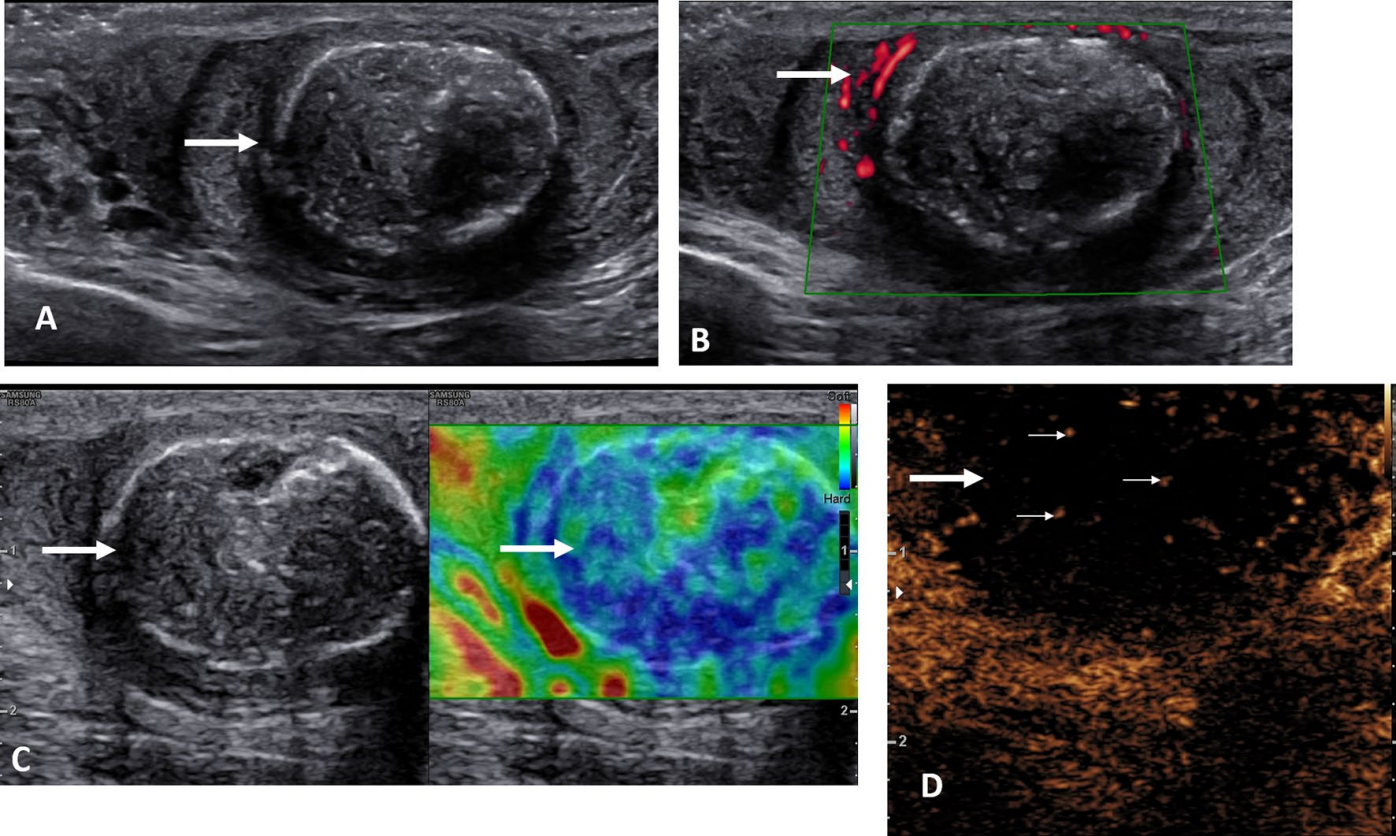
A 46-year-old with a right testicular lump, with a epidermoid cyst. **A** A longitudinal image of the right testis, with a heterogenous (arrow) occupying the central aspect of the testis, with an echogenic border, and areas of calcification. **B** The microvascular image demonstrating color flow (arrow) around the heterogenous lesion but no internal flow. **C** The tissue elastography image shows that the lesion is stiffer than the surrounding normal tissue (arrows). **D** A contrast enhanced ultrasound examination shows an avascular epidermoid cyst (large arrow) with artifact (small arrows) of high reflectivity from echogenic areas; there is no vascular flow present

**Fig. 8:**
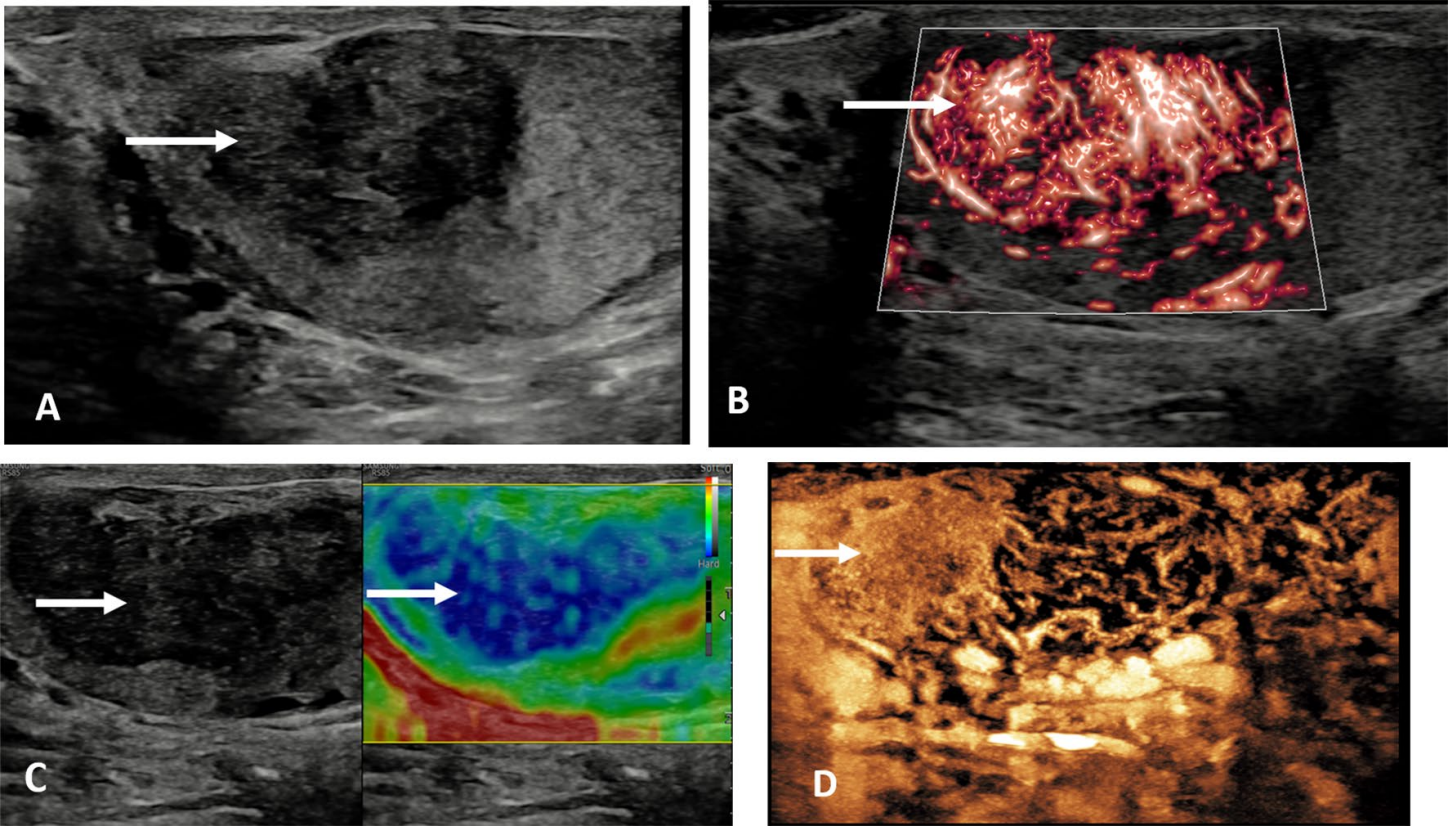
A 18-year old with known congenital adrenal hyperplasia undergoing screening ultrasound of the testis with bilateral adrenal rest cells noted. **A** Longitudinal view of the right testis on B-mode demonstrating a focal low reflective intra-testicular tumor (arrow) mimicking a primary germ cell tumor. **B** The microvascular image demonstrating color flow (arrow) within the tumor, disordered and intense in comparison to the normal testis parenchyma. **C** The tissue elastography image shows that the lesion is stiffer than the surrounding normal tissue (arrows). **D** A contrast enhanced ultrasound examination, with maximum intensity projection, demonstrates a highly vascular lesion (arrow)

The presentation of a malignant testicular tumour follows the detection of a ‘lump’ by the patient whilst self-examining, and invariably the vast majority of palpable testicular tumors are malignant germ cell tumors, classical seminomas the most common. Orchidectomy is the standard management of the large tumors, with staging by whole body CT and assessment of biochemical tumour markers [47]. Presently a number of focal testicular lesions are detected following an ultrasound for an unrelated reason, and these incidental lesions are nearly always benign, up to 80% [48]. The dilemma is to ensure that an unnecessary orchidectomy is not performed with benign disease [49], with recommendations directed towards using MPUS to ascertain the nature of the lesion to prevent this occurring [16, 50, 51]. The guidelines from EFSUMB indicate that applying CEUS and elastography to focal indeterminate lesions of the testis should be used in combination with B-mode and color Doppler techniques for a comprehensive diagnosis [11]. The current suggestion is that CEUS assessment of a malignant lesion is likely to demonstrate less intense enhancement and early washout (Fig. 5), whereas the benign lesions of Leydig cell hyperplasia (Fig. 6) are more intensely enhancing, with prolonged washout [16, 17, 52, 53]. The application of elastography as a stand-alone technique is more varied, and this has been demonstrated in recent studies with much overlap between benign and malignant lesions [7, 54], although early studies were more optimistic [55–57]. The combination of techniques is more accurate in establishing the diagnosis [58]. An example of this is the benign epidermoid cyst, a true cyst, which is avascular on color Doppler ultrasound, demonstrates no enhancement with CEUS but is of increased stiffness on elastography (Fig. 7) [59]. This is different from the rare intra-testicular adrenal rest tumors seen in congenital adrenal hyperplasia, where the focal lesion mimics a germ cell tumour, with increased vascularity, increased stiffness and marked enhancement on CEUS; a clinical history is vital (Fig. 8) [60]. Recent multi-center follow-up study on the management of the incidental discovered < 1 cm lesion may be safely followed up looking for incremental growth; >2 mm in 6 months suggests malignancy, although there is some overlap with benign lesions [61].

The rare presentation of intra-testicular lymphoma may also be interrogated with MPUS; B-mode images demonstrate low reflective lesions which may be bilateral, with increase color Doppler flow [62], with the addition of elastography demonstrating increased stiffness, and CEUS demonstrating linear non-branching vessels with early washout [63].

Intratesticular abscess formation is seen less often, and is normally a consequence of severe epididymo-orchitis, and the CEUS examination reflects the avascular components of areas of necrosis and surrounding increased enhancement form the inflamed abscess wall, with septations noted [9, 34] (Fig. 4). Chronic non-pyogenic abscesses and pseudo tumors may be difficult to diagnose with confidence on MPUS [64, 65].

### Extra testicular

**Fig. 9:**
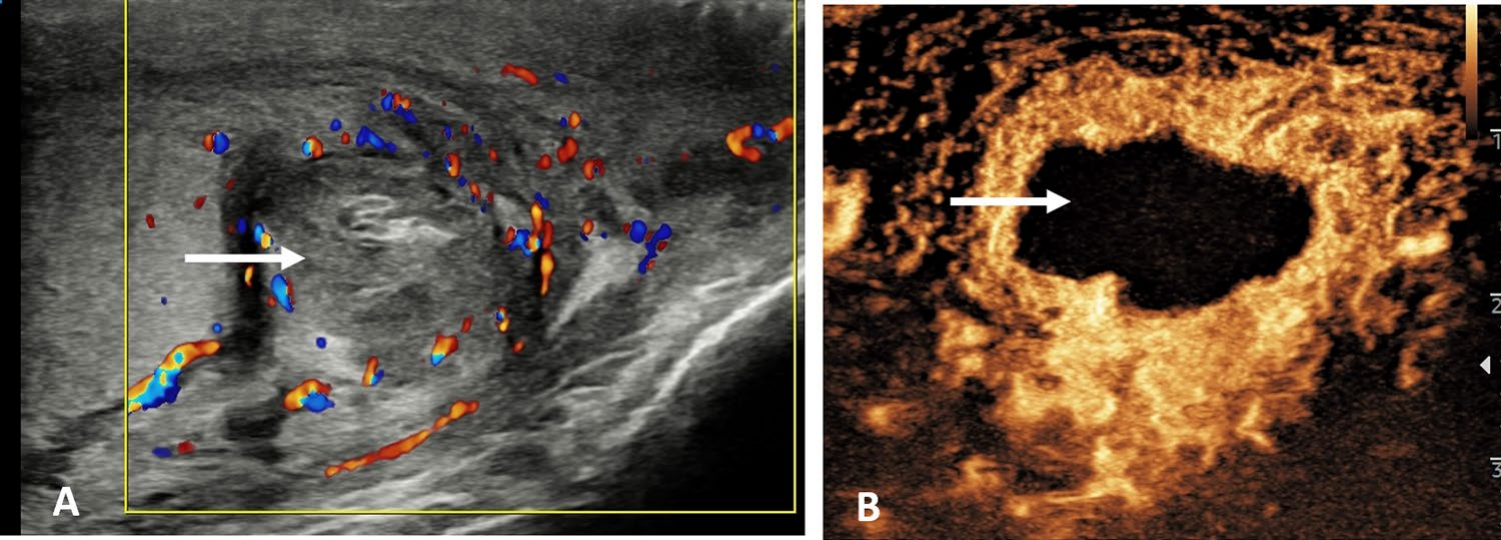
Epididymal abscess in a 56 year old. **A** Longitudinal view of the right testis demonstrating an echogenic lesion in the epididymal tail (arrow)in epididymitis, with surrounding increase in color Doppler signal. **B** A contrast enhanced ultrasound examination demonstrates no vascularization in the center of the lesion (arrow) with increased contrast enhancement surrounding the avascular cavity confirming an epididymal abscess

**Fig. 10:**
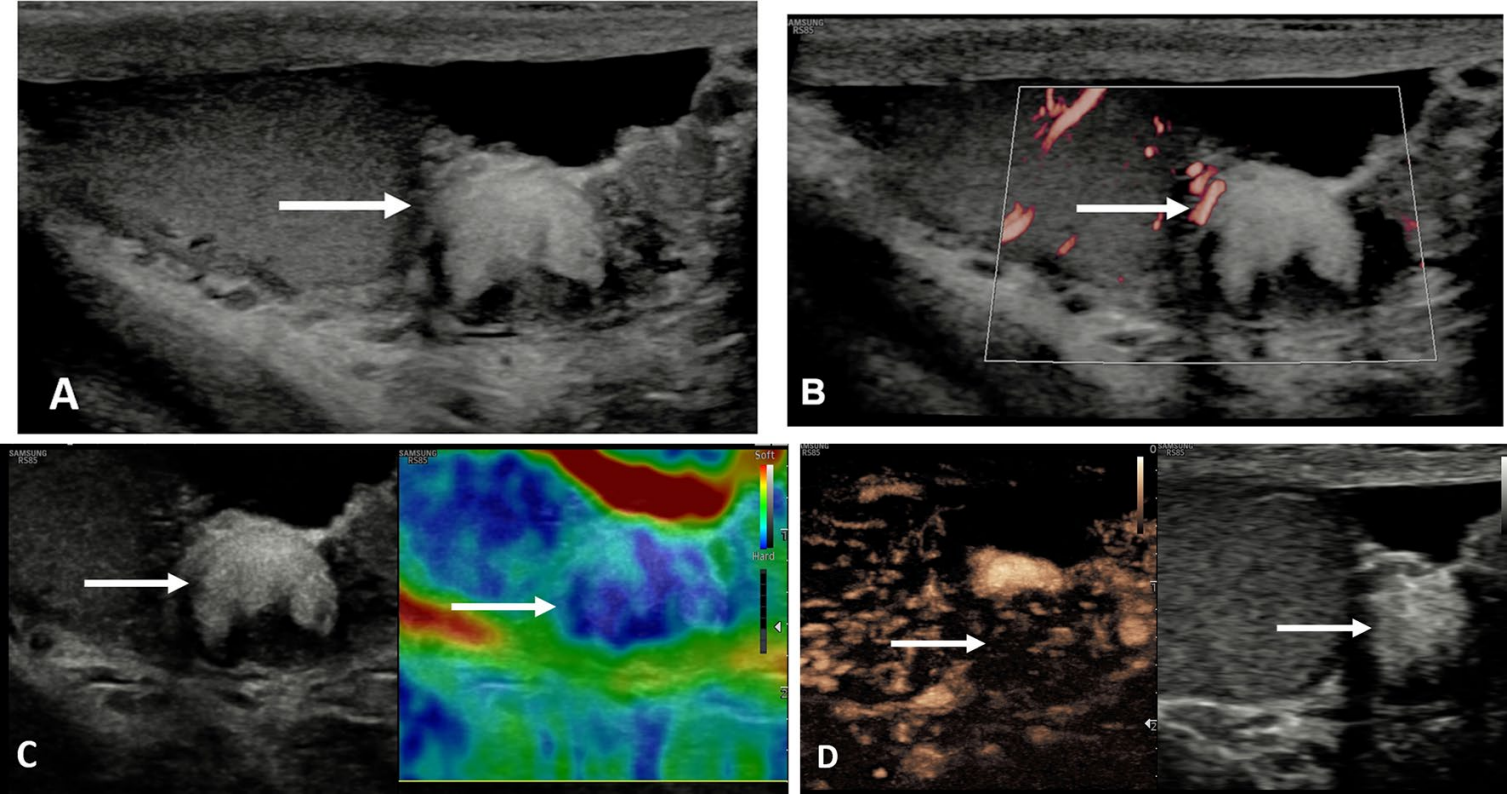
Palpable mass in the left epididymis in a 48-year old, with characteristics of a benign lipoma. **A** A longitudinal B-mode image of the left testis demonstrating an echogenic lesion in the epididymis (arrow). **B** Microvascular imaging demonstrates some vascular flow in the periphery of the lesion, but no central flow (arrow). **C** On elastography the echogenic lesion demonstrates increased stiffness (arrows). **D** On the contrast enhanced ultrasound examination, internal microbubble signal is observed (arrow) indicating some vascularization of the lipoma

In the adult nearly all extra-testicular abnormalities are benign, with epididymal abscess formation (Fig. 9) following infection the most common painful focal lesion, and with painless lesions most commonly cystic or a spermatocele, and with solid lesions likely a lipoma (Fig. 10) or an adenomatoid tumour [66, 67]. The application of MPUS in extra-testicular lesions adds information, with B-mode findings alone nearly always diagnostic. Rarely is an extra-testicular lesion in the adult a malignant rhabdomyosarcoma or a mesothelioma, with presentation as a painless mass [68]. In children, an extra-testicular lesion is often a rhabdomyosarcoma and warrants urgent investigation.

### Inguinal lesions

The presence of an undescended inguinal testis can be documented in adult patients and an ultrasound examination is often confirmatory. The addition of color Doppler techniques will confirm viability of the testis, and addition of CEUS in patients with recent pain, establishes the possibility of vascular compromise [69]. An inguinal spermatic cord mass in an adult is rare, and abnormalities of the spermatic cord may arise at this site, sometimes benign, a cavernous hemangioma or with leiomyosarcomas reported [70–73]. The presence of abnormal color Doppler flow alerts the examiner to possibility of malignancy, with increased stiffness on elastography a hallmark of malignancy [74].

## Trauma

Trauma to the scrotal sac is invariably a result of a sporting injury or a motor vehicle injury in a young man [75]. The contents of the scrotal sac may be involved, with testicular fracture and rupture most concerning, with an intact tunica albuginea essential to testicular viability; rupture requires surgical correction [76, 77]. Epididymal rupture and tears also occur and often require surgical management [78, 79]. An isolated intra-testicular hematoma may ensue, and may also be iatrogenic from a testicular biopsy procedure.

### Intra-testicular hematoma

**Fig. 11:**
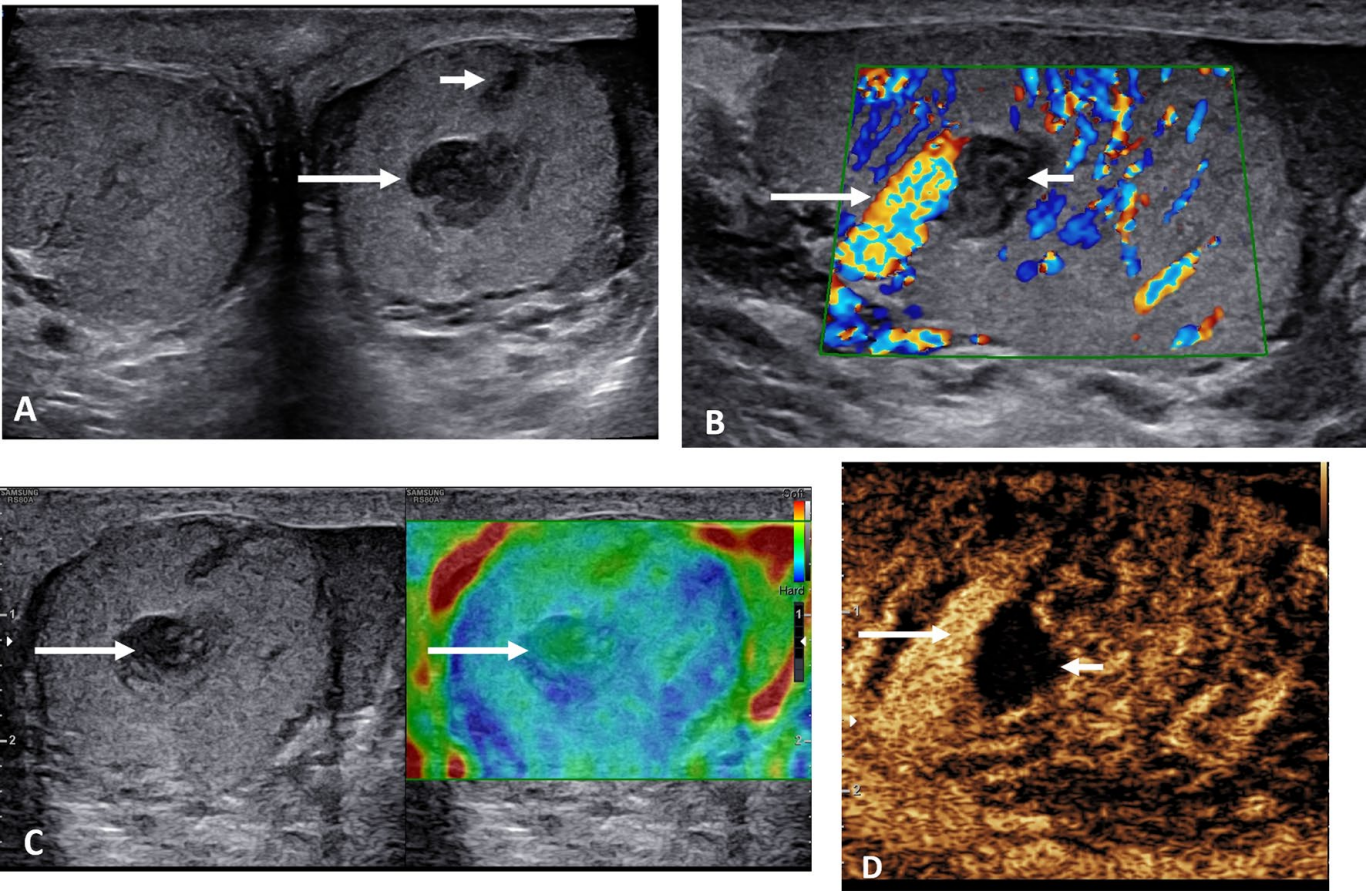
Post-biopsy pain, biopsy performed for investigation of infertility in a 45 year old, with an intra-testicular hematoma and arterio-venous fistula. **A** A transverse image on B-mode, demonstrating a focal low reflective lesion in the mid aspect of the left testis (long arrow). The biopsy tract is seen as a linear low reflective area (short arrow). **B** An oblique color Doppler ultrasound showing the presumedhaematoma (short arrow), with increased flow (long arrow) in a arterio-venous fistula. **C** On elastography the stiffness is intermediate (arrows), and will evolve with the stage of the liquefication of the hematoma. **D** On the contrast enhanced ultrasound examination, the hematoma (short arrow) is clearly depicted, with the arterio-venous fistula obvious (long arrow)

The presence of a hematoma is readily identified on an ultrasound examination, and is often seen in the context of recent blunt scrotal trauma [80]. With trivial or forgotten trauma, confusion with a primary germ cell tumour is a possibility, and the importance of MPUS in establishing the benign nature of the lesion, allowing conservative management is recognized [81]. The ability of a CEUS examination to exclude the presence of vascularity in the lesion, and the use of elastography to exclude increased stiffness, allows for serial examinations to demonstrate regression of the lesion [82, 83]. This avoids an unnecessary orchidectomy. Following testicular biopsy, usually for investigation of infertility, monitoring a complicating hematoma with MPUS, alleviates both patient and physician anxiety (Fig. 11).

### Testicular fracture

The B-mode ultrasound often depicts the site of rupture of the tunica albuginea, with herniation of testicular tissue, allowing for targeted surgical repair. The viability of the underlying testis on color Doppler US alone is not conclusive, and adding a CEUS examination clearly depicts the viable perfused testicular tissue [84]. A CEUS examination could dictate the possibility of a partial orchidectomy to preserve viable testicular tissue and future fertility prospects [85].

### Epididymal trauma

B-mode ultrasound will evaluate the epididymis in trauma with the addition of color Doppler ascertaining the vascularity. Addition of CEUS allows for the identification of a hematoma, extent of the epididymal injury and will allow for confident management [75].

## Testicular microlithiasis

**Fig. 12:**
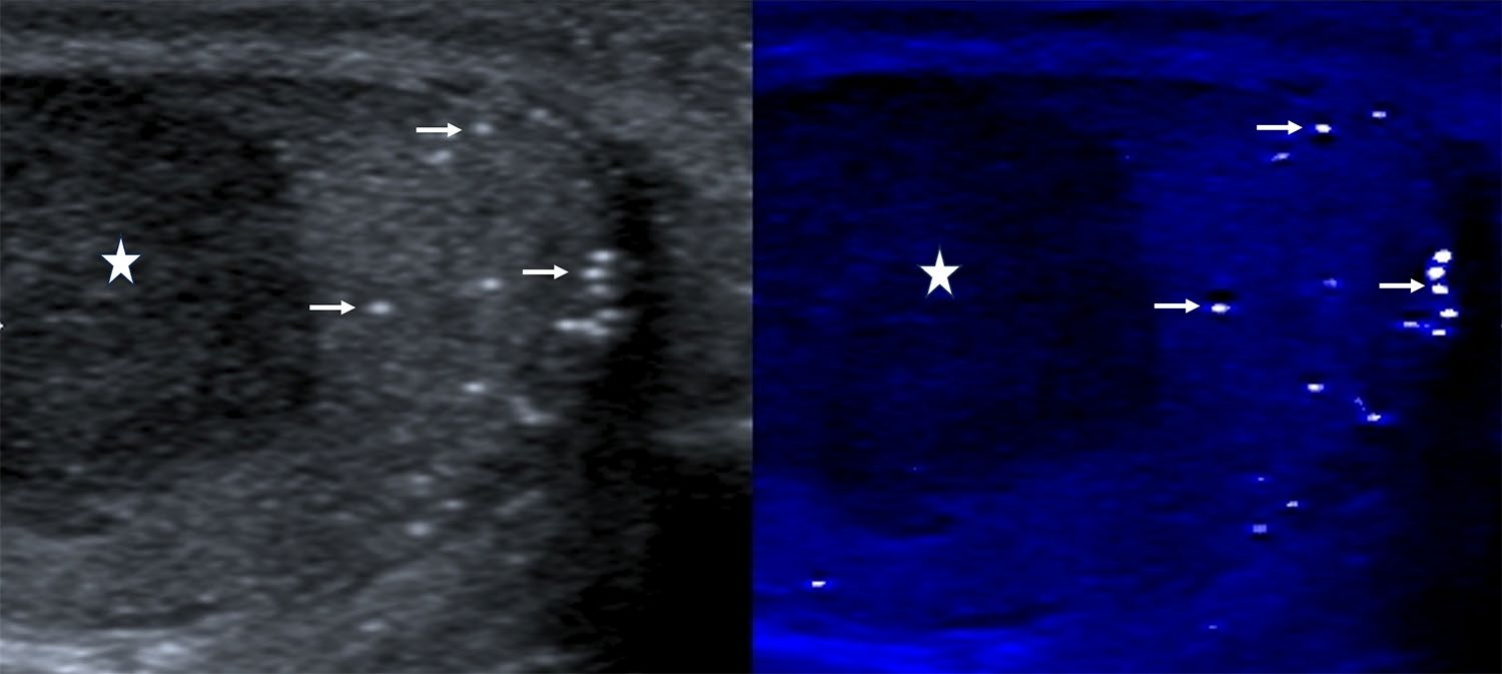
Longitudinal images of the left testis, with classical microlithiasis. The MicroPure™ (Canon Medical Systems) demonstrates the areas of microlithiasis within the testis (arrows) with background suppression, making the testicular microlithiasis more obvious. A primary germ cell tumor is present (star)

There has been extensive interest in the epidemiology of testicular microlithiasis and the association with primary germ cell tumors of the testis, with debate on the association and the need for ultrasound surveillance [86–88]. Studies on the application of elastography in testicular microlithiasis have demonstrated slight increase in stiffness compared with the normal testis, but less than in the presence of a germ cell tumor [89]. There is no role for color Doppler ultrasound in testicular microlithiasis. Areas of macrocalcification are occasionally encountered, may be associated with a higher prevalence of tumors that microlithiasis [90], and may demonstrate the “twinkle” artifact on color Doppler ultrasound [91]. The B-mode appearances are well documented, and subtle areas of limited testicular microlithiasis may be better depicted using a technique that accentuates areas of calcification, more often used for subtle calcification seen on breast ultrasound (Fig. 12).

## Conclusion

The addition of the newer ultrasound techniques, including elastography and CEUS add another dimension to the evaluation of scrotal diseases. The ability to ascertain the presence or absence of vascularity on a CEUS examination is crucial in establishing the viability of the testicular or epididymal tissue, identifying a hematoma or abscess or establishing a infarction. Assessment of the pattern of enhancement of a neoplastic lesion of the testis can differentiate the benign from the malignant neoplastic testicular lesion, allowing testis sparing surgery, watchful waiting and in the clinical context, preserves fertility [92]. The combination with elastography gives added information, with a caveat that no US technique should be used as a stand-alone imaging technique but the combination will yield a more comprehensive outcome in terms of diagnosis and management both in the adult and pediatric patient [9, 93].

## Data Availability

No datasets were generated or analysed during the current study.
